# Adrenal Cystic Lymphangioma: An Unexpected Pathological Finding in a Constellation of Uncontrolled Hypertension and Hypercalcemia

**DOI:** 10.7759/cureus.5741

**Published:** 2019-09-24

**Authors:** Jad A Degheili, Gerges D Bustros, Jose El-Asmar, Nassib Abou Heidar, Rami W Nasr

**Affiliations:** 1 Department of Surgery, Division of Urology, American University of Beirut Medical Center, Beirut, LBN

**Keywords:** adrenal cysts, lymphangioma, pheochromocytoma, hypertension

## Abstract

Adrenal cysts are rarely observed lesions. Adrenal cystic lymphangiomas are asymptomatic benign lesions of the lymphatic vessels with the vast majority occurring in women. We herein present a rare case of a middle-aged gentleman with labile blood pressure associated with an incidental finding of an adrenal mass of 4 x 3 x 3 cm. Following surgical resection, pathology revealed the diagnosis of adrenal cystic lymphangioma.

## Introduction

Adrenal cysts are rare lesions categorized into four different groups: adrenal endothelial cysts (45%), pseudocysts (39%), epithelial cysts (9%), and parasitic cysts (7%) [1]. Most of these adrenal cystic lesions are asymptomatic and are either discovered incidentally upon imaging studies or during surgery for an unrelated complaint [2]. Adrenal endothelial cysts are further classified into hemangiomas, hamartomas, and Lymphangiomas [1]. Adrenal cystic lymphangiomas (AL) are benign lesions of the lymphatic vessels with the vast majority of cases reported in women [3]. To the authors’ best knowledge, there have only been two female cases with AL associated with hypertension. Herein, we present a case of an AL in a middle-aged male who presented initially with uncontrolled hypertension and hypercalcemia. 

## Case presentation

**Figure 1:**
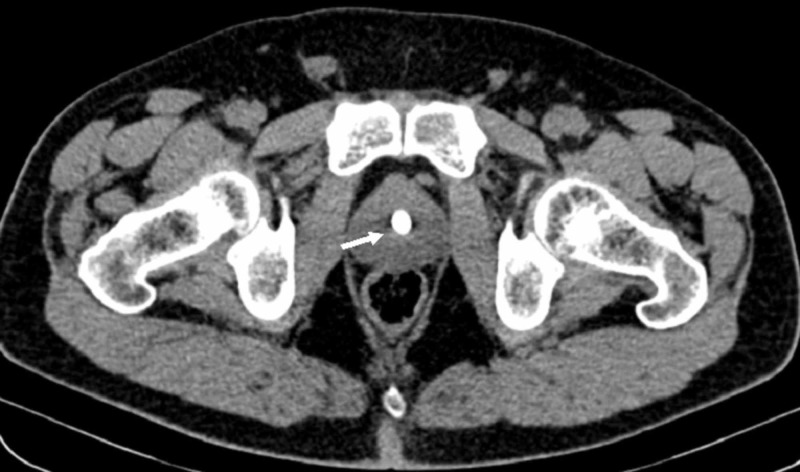
Unenhanced CT scan of the pelvis (axial view) showing the presence of a 9-mm calculi within the prostatic urethra CT, computed tomography

A 53-year-old gentleman, previously healthy, presented to the emergency department with a three-day history of right flank pain along with dysuria and elevated systolic blood pressure of 200 mmHg. He denies a recent history of fever, chills, or any gastrointestinal symptoms. Initial workup showed a normal white blood cell count and a serum creatinine level of 1.3 mg/dL; yet, an elevated calcium level of 11.7 mg/dL (8.5-10.1 mg/dL). A non-enhanced CT scan showed the presence of a 9.5-mm stone within the prostatic urethra (Figure 1).

**Figure 2:**
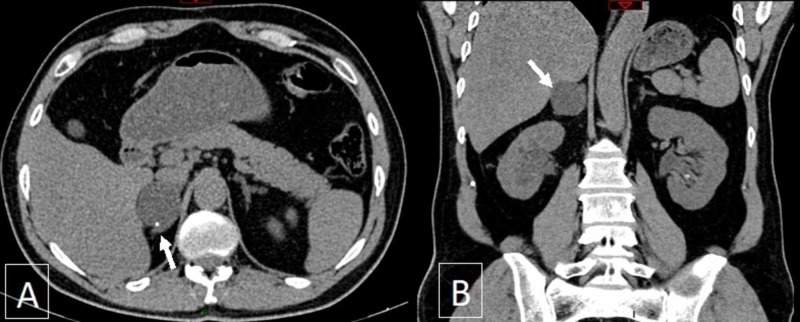
Plain CT scan of the abdomen and pelvis showing a well-defined and rounded cystic lesion arising from the median limb of the right adrenal gland (arrow), measuring 4.0 x 3.7 x 3.4 cm with a fluid density of 12 HU A small calcification is seen at its rim (arrowhead). A: axial view. B: coronal view. CT, computed tomography; HU, Hounsfield unit

A right suprarenal cystic mass measuring 4.0 x 3.7 x 3.4 cm was incidentally found to be arising from the median limb of the right adrenal gland, with rim calcification (Figure 2).

The measured Hounsfield unit (HU) for this lesion was 12. Since there were neither signs of infection nor acute kidney injury, the elevated blood pressure took precedence over the documented urethral stone, and hence, further work-up of this adrenal mass was done before an intervention for the prostatic urethral stone. 

Additional blood and urine tests revealed an elevated serum parathyroid hormone (PTH) level of 181 pg/mL (11-55 pg/mL) along with an elevated serum nor-metanephrine level of 298 ng (less than 170 ng/L) and a metanephrine level of 169 ng/L (less than 73 ng/L)). A 24-hour urine collection also revealed an elevated nor-metanephrine level of 915 µg per 24 hours (0-600 ug per 24 hours) and a metanephrine level of 423 µg per 24 hours (0-350 µg per 24 hours). Moreover, the serum aldosterone level was 524 pg/mL (38-146 pg/mL), Renin level was 19 mIU/L (2.8-40 mIU/L), and the aldosterone/ renin ratio was also elevated with a value of 73 (less than 64).

**Figure 3:**
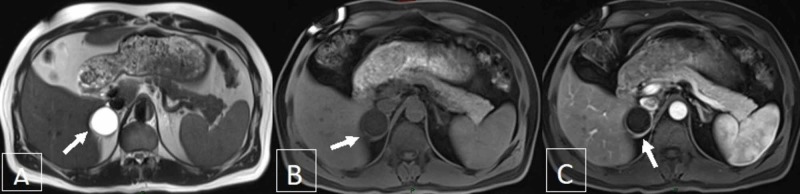
MRI of the abdomen and pelvis with gadolinium (A) right adrenal cystic lymphangioma lesion (arrow) as evident on T2 sequence, axial view, measuring around 4.0 x 3.7 cm with homogeneous signal intensity; (B & C) T1 sequence, pre- and post-contrast phase, respectively, showing the same cystic lymphangioma lesions, arising from the right adrenal gland, exhibiting rim enhancement (arrow) with absence of solid or fat component MRI, magnetic resonance imaging

For better characterization of the incidental adrenal mass, MRI of the abdomen with gadolinium was performed revealing this same size adrenal mass with homogeneous signal intensity, mild enhancement of its borders, and with the absence of any fat or solid component (Figure 3).

The combination of clinical and laboratory findings consisting of an adrenal mass alongside an elevated systolic blood pressure was suggestive of a functionally secreting adrenal mass, resembling most likely a pheochromocytoma. Furthermore, hypercalcemia in such a setting suggested the presence of either a parathyroid hyperplasia or adenoma; the presence of later was confirmed by ultrasound and (99m) Tc sestamibi scintigraphy scan apriori. All those findings contributed to a possible constellation of multiple endocrine neoplasia (MEN) 2A syndrome. Consequently, the patient was started on a combination of an alpha and a beta blocker, more specifically Doxazocin and Bisoprolol in preparation for definitive surgery.

**Figure 4:**
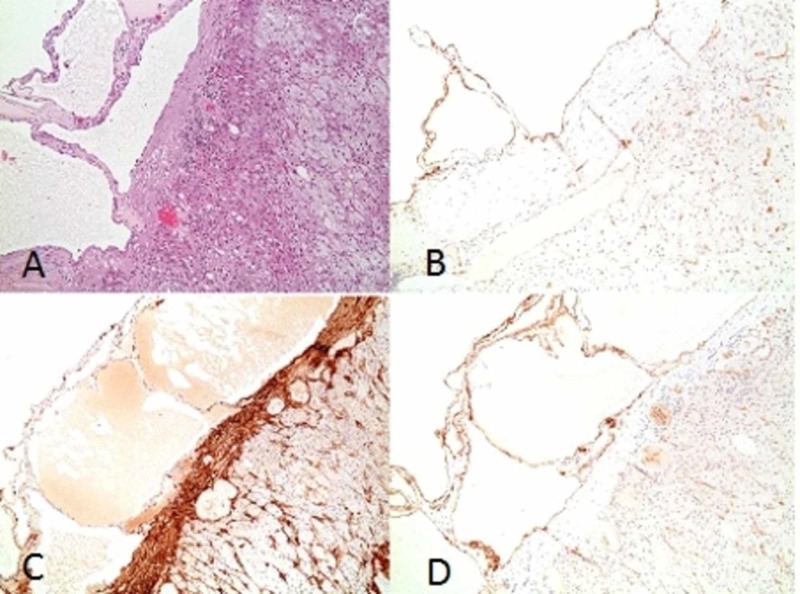
A: Hematoxylin & Eosin stain showing multilocular cyst lined by single layer of flattened endothelial cells and thin fibrous septa that focally contained some lymphocytes; no atypia is noted; B & D: Immunostaining on cross-section showing cells lining the cystic spaces positive for CD31; C: Immunohistochemistry showing positive SMA staining CD, cluster of differentiation; SMA, smooth muscle actin

After achieving a tight blood pressure control, a right open adrenalectomy through a trans-peritoneal subcostal incision was done. Grossly, an 8 x 6.5 x 2.5 cm adrenal gland weighing 35 g was resected without any complications. Upon pathological and histological studies, cross- sectioning of the specimen revealed a 3 x 3 x 2-cm cyst that is filled with clear gelatinous and proteinaceous material with the absence of any solid intracystic excrescences. Thorough examination of the cyst revealed its multilocular nature with a wall thickness ranging from 1 to 3 mm forming a single layer of flattened endothelial cells. The spaces between the small cysts were detached by a fibrous septa rich in lymphocytes. Adrenal hyperplasia, adrenal adenoma, adrenal cortical carcinoma, or pheochromocytoma were ruled out. Immunohistochemistry staining of the cells lining the cystic spaces were positive for CD31 and SMA, but negative for CD34 (Figure 4).

The diagnosis was consistent with an adrenal cystic lymphangioma. The patient had an unremarkable post-operative course and was discharged home on post-op day 5. On his one-month follow-up, he reported adequate blood pressure control without the use of any antihypertensive medication.

Later, a parathyroidectomy was performed for the left inferior parathyroid adenoma and pathology came out to be consistent with an adenoma measuring 2.5 x 2.5 x 0.5 cm. The urethral stone was then fragmented at a later stage, endoscopically using holmium laser.

## Discussion

Adrenal cysts are rare entities with a low prevalence rate. According to autopsy studies, they range between 0.064% and 0.18% [4]. The peak incidence of presentation is between the third and the sixth decade of life, yet may present at any age. A female predominance is noted with a ratio of 2:1 compared to males [4]. They are usually unilateral without a preference favoring either side [4].

Lymphangiomas are benign lymphatic lesions occurring throughout the body preferentially in the neck, axilla, and mediastinum. Only 5% occur in the abdomen and pelvis [5]. The majority of reported intra-abdominal lymphangiomas are located in the mesentery with only a few reported cases presenting in the adrenal glands. The literature reports a total of 53 cases of adrenal lymphangiomas to date.

Four histological subtypes have been described for ALs: cystic, capillary, cavernous, and vasculolymphatic malformation; the latter described as the presence of endothelial lined lymphatic channels separated by connective tissue [6]. The pathogenesis of AL is still unclear and debatable, yet the most acceptable theory emphasizes on an abnormal development leading to lymphatic vessel ectasia [5].

The most common presentation of AL is an incidental finding upon investigation for an unrelated complaint. AL can develop into a large size while remaining asymptomatic [2]. Symptomatic patients with AL may witness fever and/or abdominal pain with distention [2].

The initial presentation of the significantly elevated blood pressure along with an incidental finding of an adrenal mass, albeit cystic in nature, did, however, raise the suspicion of a pheochromocytoma. Cystic pheochromocytomas are rare findings compared to its solid counterpart and have been described in case reports only [7-8]. Their pre-operative diagnosis is very difficult, as clinical, biochemical, and radiological findings especially those on the MRI may not be consistent with the usual pheochromocytomas [9]. It is postulated that the cystic nature of such lesions is due to hemorrhagic degeneration, necrosis, and cyst formation [9]. Careful surgical excision is needed to avoid spillage of fluid that contains high levels of catecholamines and metanephrines [10]. 

Three different imaging modalities contribute to the characterization of adrenal cystic lymphangiomas. Ultrasonography shows a well-marginated and anechoic lesion; MRI shows a low-intensity signal on T1-weighted images and high-intensity signals on T2-weighted images, CT scan shows a non-enhancing hypodense lesion [11]. Nevertheless, final diagnosis can be only achieved by surgical resection followed by pathologic confirmation. 

If the suspicion for malignancy is low, some authors have recommended aspiration of the adrenal cyst as a diagnostic and therapeutic approach [6]. However, a consensus for surgical resection of the adrenal cyst is recommended with any of the following features: large mass, parasitic content, the potential for malignancy, or symptomatic presentation.

Finally, someone may posit the hypothesis of a certain syndrome, given the presence of an adrenal mass along with a functioning parathyroid adenoma, as a cause of hypertension and hypercalcemia. The first that comes to mind is MEN syndrome. To date, no cases of adrenal cystic lymphangioma with MEN syndromes have been reported. Even the newly discovered and rarest MEN4 syndrome, secondary to cyclin-dependent kinase inhibitor 1b (CDKN1B) gene mutation, did not possess adrenal cystic lymphangioma in its constellation of findings so far [12]. Nineteen cases of MEN4 have been reported up to date, with the following various manifestations: pituitary tumors, acromegaly, Cushing disease, primary hyperparathyroidism, papillary thyroid carcinoma, adrenal tumors, and various lung, bronchial, gastrointestinal neuroendocrine tumors (NET) [13]. An interesting observation in that series is that almost all cases of MEN4 exhibit hyperparathyroidism as a clinical manifestation. Similar to all other MEN syndromes, genetic testing for all affected patients and families is essential for establishing diagnosis. 

## Conclusions

In summary, a 53-year-old gentleman presented with a clinical picture of an adrenal mass associated with emergency hypertension, borderline elevation of endocrine markers, and hypercalcemia. Upon surgical resection of the adrenal mass, he was found to have adrenal cystic lymphangioma, contrary to our anticipation. All symptomatic adrenal cysts must be approached surgically; adrenal cystic lymphangioma is a rare entity that should be included in the differential diagnosis of adrenal cysts.

## References

[1] Michalopoulos N, Laskou S, Karayannopoulou G, Pavlidis L, Kanellos I (2015 Dec). Adrenal gland lymphangiomas. Indian J Surg.

[2] Ellis CL, Banerjee P, Carney E, Sharma R, Netto GJ (2011 Jul). Adrenal lymphangioma: clinicopathologic and immunohistochemical characteristics of a rare lesion. Hum Pathol.

[3] Joliat GR, Melloul E, Djafarrian R (2015). Cystic lymphangioma of the adrenal gland: report of a case and review of the literature. World J Surg Oncol.

[4] Longo JM, Jafri SZ, Bis KB (2000 Mar-Apr). Adrenal lymphangioma: a case report. Clin Imaging.

[5] Ting-Po L, Marcelo C, Chi-Kuan C, Jong-Ming H, Wun-Rong L (2014). Adrenal cystic lymphangioma: a case report and review of the literature. Urologic science.

[6] Ates LE, Kapran Y, Erbil Y, Barbaros U, Dizdaroglu F (2005). Cystic lymphangioma of the right adrenal gland. Pathol Oncol Res.

[7] Cajipe KM, Gonzalez G, Kaushik D (2017. pii). Giant cystic pheochromocytoma. BMJ Case Rep. 2017 Nov 8.

[8] Gupta A, Bains L, Agarwal MK, Gupta R (2016 Jul-Sep). Giant cystic pheochromocytoma: a silent entity. Urol Ann.

[9] Junejo SZ, Tuli S, Heimann DM, Sachmechi I, Reich D (2017 Jul). A case report of cystic pheochromocytoma. Am J Case Rep.

[10] Costa Almeida CE, Silva M, Carvalho L, Costa Almeida CM (2017). Adrenal giant cystic pheochromocytoma treated by posterior retroperitoneoscopic adrenalectomy. Int J Surg Case Rep.

[11] Geramizadeh B, Yazdanpanah S, Salahi H, Marzban M (2015). Adrenal cystic lymphangioma presented with hypertension: a case report. Nephrourol Mon.

[12] Lee M, Pellegata NS (2013). Multiple endocrine neoplasia type 4. Front Horm Res.

[13] Alrezk R, Hannah-Shmouni F, Stratakis CA (2017 Oct). MEN4 and CDKN1B mutations: the latest of the MEN syndromes. Endocr Relat Cancer.

